# Biocatalytic reduction of nitro-sulfonamides: enzyme engineering, process integration, and sustainable pathways to *p*-aminobenzenesulfonamide

**DOI:** 10.3389/fbioe.2026.1867973

**Published:** 2026-07-20

**Authors:** Rui Ai, Noor Muhammad, Yuelong Cui, Liqiang Wang, Yao Wang, Shaoting Weng, Mohib Ullah Kakar, Suliman Khan, Sajjad Ahmad

**Affiliations:** 1 College of Biology and Food Engineering, Anyang Institute of Technology, Anyang, China; 2 College of Life Sciences, Jinggangshan University, Ji’an, China; 3 Biomedical Sciences, Radiation Therapy Department, Anyang District Hospital, Puyang, China; 4 Faculty of Marine Sciences, Lasbela University of Agriculture Water and Marine Sciences (LUAWMS), Uthal, Balochistan, Pakistan; 5 Faculty of Veterinary and Animal Sciences, Lasbela University of Agriculture, Water and Marine Science, Uthal, Baluchistan, Pakistan

**Keywords:** biocatalysis, cofactor regeneration, nitroreductase (NTR), nitro-sulfonamide reduction, sustainable process integration

## Abstract

Sulfonamides remain important to medicinal and fine-chemical production but conventional aniline precursor routes like Pd-catalysed hydrogenation and Béchamp reductions carry sustainability, safety, and chemoselectivity challenges, especially for N-S bond integrity. This review assesses biocatalytic nitroreduction as a selective alternative for nitro-sulfonamides, focusing on *p*-aminobenzenesulfonamide (*p*-ABS). We summarise mechanistic and engineering advances in Type I (oxygen-insensitive) flavin-dependent nitroreductases (NTRs), highlight emerging roles and limits of Old Yellow Enzymes, and discuss auxiliary reductive platforms (H_2_-driven hydrogenases, photo-/electro-biocatalysis) for improved cofactor economy and endpoint selectivity. Process-intensification strategies, whole-cell vs. cell-free operation, immobilisation, packed-bed flow, on-line LC/IR PAT, and NAD(P)H regeneration via GDH/FDH or electroenzymatic modules are mapped to chemoselectivity risks (hydroxylamine accumulation, azo/azoxy formation) and mass-transfer constraints. We highlight development choices with green metrics (PMI, E-factor) and emphasise early LCA integration to avoid burden shifting from buffer salts or mediator residues. Evidence from continuous NTR reactors and immobilised formats supports scalable, aqueous, low-pressure operation; however, direct data on sulfonamide-linked nitroarenes is limited. This motivates a feasibility screen using an NTR panel (including engineered NfsB lineages), water-rich media with low co-solvent, and O_2_-tolerant settings. We conclude with a proposed flowsheet for *p*-ABS coupling immobilised NTR with FDH or electro-NAD(P)H supply, real-time analytics, and membrane-based product extraction. As direct data on sulfonamide-linked nitroarenes remain limited, this roadmap provides a practical and critical starting point for substrate-specific feasibility screening and future development.

## 1 Introduction

Sulfonamides remain important for medicinal and fine chemical synthesis, with persistent relevance spanning anti-infectives and enzyme inhibitors. Their prototypical core, *p*-aminobenzenesulfonamide (sulfanilamide), started the antibiotic era and still features as a very important element in discovery campaigns and derivative synthesis. This keeps their demand for finding robust, selective and sustainable access routes. Recent studies suggest the historic centrality and ongoing diversification of sulfonamide chemistry and applications, ranging from DHPS-targeting antibacterial drugs to diuretic, hypoglycemic, antiglaucoma, protease and kinase inhibitory, and anti-inflammatory series. These studies also report modern approaches to S-N bond formation and point out the importance of greener transformations at all stages of the sulfonamide life-cycle (Chhablani et al., 2025; Noor et al., 2025).

Industrial production of anilines and related aryl amines such as sulfonamide precursors has long relied on catalytic hydrogenation of nitroarenes over supported precious metals (for example, Pd/C), and historically, the Béchamp reduction (iron/acid). Both approaches can deliver high conversions. However, sustainability and safety concerns exist. Béchamp processes generate large quantities of iron-bearing sludge and require further effort, besides several innovations (like recent mechanochemical variants) which are intended to cut solvent and waste footprints (Guo et al., 2025; Kubota et al., 2025). In comparison, Pd-catalysed hydrogenations are involved in multipurpose manufacturing, yet remain vulnerable to sulfur-induced deactivation and complex selectivity management when sulfur-containing nitroarene substrates or feeds with high S-impurities are present, a significant issue for nitroaromatics. Contemporary catalysis research continues to engineer around sulfur poisoning and to refine mechanisms of hydrogen spillover and active-site regulation. Nonetheless; catalyst stability, metal use and hydrogen handling keep the environmental and operational costs significant (Zhang X. et al., 2022; Zhang et al., 2023; Ren et al., 2025; Sun et al., 2025).

Chemoselectivity is a critical factor when handling sulfonamides along with catalyst life. This is important especially under reductive conditions where stable sulfonyl protecting groups are vulnerable to cleavage. As selective removal of other functionalities (for example, halogens) may be desired, inadvertent desulfonylation pathways can compromise yields and disrupt downstream protection strategies. Recent photocatalytic desulfonylation methods have also reported this phenomenon. Accordingly, approaches that minimize the driving forces for N-S bond cleavage while effectively targeting alternative functional group removal are highly valuable (Corpas et al., 2022; Pavlovska et al., 2023).

Biocatalytic nitroreduction has emerged as a selective alternative, against this problem. Flavin-dependent nitroreductases (NTRs), particularly oxygen-insensitive enzymes like those from *Enterobacter cloacae*, catalyze two-electron reductions of nitroaromatics to nitroso and hydroxylamine intermediates using NAD(P)H as the reductant, and has been reported in kinetic studies. Recent reports show that NTRs can act on many different nitroaromatic compounds because they use a simple “ping-pong” reaction mechanism without any gate, as seen with para-nitrobenzoic acid. This catalytic versatility supports their application in bioremediation of nitroaromatic pollutants, with potential for synthesizing nitrogen-containing compounds such as azoxy compounds and N-heterocycles (Pitsawong et al., 2014; Russo et al., 2025).

Other mechanistic reports and reactivity discovery reveal that Old Yellow Enzymes (OYEs), traditionally recognized as canonical ene-reductases, exhibit variable transformations. In the Morita-Baylis-Hillman (MBH) reaction, they enable the adaptation of these flavoenzymes for non-native C-C bond-forming reactions. Mechanistic studies using GkOYE with 4-nitrobenzaldehyde as a substrate, alongside engineering efforts to develop GkOYE.8, GkOYE.11, and GkOYE.13, demonstrate the way native reduction functions can be modified or enhanced to catalyze the MBH reaction, producing (*R*)- and (*S*)-MBH adducts with improved efficiency and stereoselectivity (Sahrawat et al., 2024; Wang L. et al., 2024).

The biocatalytic processes utilize enzyme systems in cell-free formats with immobilized catalysts, including hydrogenase on carbon black and reductive graphene quantum dots (rGQDs) coupled with aldo-keto reductase (AKR). These systems facilitate efficient reductions of nitro compounds and prochiral ketones under mild, aqueous conditions, with the hydrogenase system achieving full conversion of 30 nitroarenes (isolated yields 78%–96%) using 1 bar H_2_, and the rGQDs/AKR system yielding 82% (*R*)-3,5-bis(trifluoromethyl)phenylethanol ((*R*)-3,5-BTPE) with >99.99% enantiomeric excess under infrared (IR) illumination. The hydrogenase system delivers electrons from H_2_ to a carbon support for selective nitro group reduction, while rGQDs enable water-mediated reductions by splitting water under IR light, both eliminating nicotinamide cofactor dependency. The hydrogenase system demonstrates tolerance to a wide range of functional groups, stability in reactions lasting up to 72 h, and reusability over five cycles with a turnover number exceeding one million. The rGQDs/AKR system exhibits high enantioselectivity, validated by molecular simulations showing a preference for the pro-(*R*) binding form of 3,5-BTAP (Sokolova et al., 2024; Wang et al., 2025).

These scientific and technological shifts align with a sustainability framework for route selection in pharma and fine chemicals. Dedicated analyses of biocatalysis waste streams emphasize the need for explicit green-metric accounting, such as E-factor, process mass intensity (PMI) along with transparent discussion of primary and secondary waste (for example, wastewater, organic fractions), and life-cycle impacts. These analyses highlight the need for realistic assessments that include the environmental impact of waste treatment processes (Domínguez de María, 2025; Handa et al., 2025).

Keeping in view the market, the case for biocatalytic access to *p*-aminobenzenesulfonamide is reinforced by sustained medicinal interest in amino-benzenesulfonamide derivatives and their upstream intermediates across oncology, antimicrobial agents and enzyme inhibition. Increasing reports of sulfonamide-based series and terminal derivatisations show an expanding space where clean aniline installation and protection-group integrity are both critical. Features that are aligned with biocatalytic chemoselectivity (Mancuso et al., 2022; Elsayad et al., 2025; Sharif et al., 2025).

Despite the considerable promise of nitroreductases (NTRs) for the chemoselective reduction of nitroaromatic and nitroheterocyclic compounds to anilines under mild, hydrogen-free conditions (Ruiz et al., 2025; Bisagni et al., 2022), two practical gaps exist. First, while NTRs have been broadly profiled across diverse nitroarene scaffolds, systematic data on nitro-sulfonamides, and specifically the biocatalytic conversion of *p*-nitrobenzenesulfonamide (*p*-NBS) to *p*-aminobenzenesulfonamide (*p*-ABS, sulfanilamide) remain limited. Although a few studies have reported partial activity with selected NTRs or engineered variants (Park et al., 2015). Some valuable substrate-specific information has been reported through the evaluation of a panel of fourteen nitrobenzene and four nitropyridine derivatives using four nitroreductases (NR-04, NR-14, NR-17, and NR-24). These have been produced in *E. coli* as lyophilized cell extracts (Bisagni et al., 2022). A broader benchmarking of enzyme classes, expression hosts, media conditions, and cofactor-regeneration strategies against this industrially important family of electron-deficient nitroaromatic compounds remains highly beneficial. Second, enabling technologies such as cofactor recycling (glucose/GDH-101 system), fed-batch operation, and co-solvent tolerance (up to 20% v/v toluene, MTBE, or DMSO) have advanced considerably. Consolidated process-integration guidance addressing cofactor economy, oxygen management for O_2_-tolerant variants, solvent selection, immobilisation, continuous-flow operation, and real-time PAT has not yet been assembled specifically for nitro-sulfonamide targets.

In this study we (i) discuss biocatalytic nitroreduction as a selective, scalable and sustainability-aligned alternative to classical Pd/H_2_ and Béchamp routes for nitro-sulfonamides; (ii) utilize the latest insights in enzyme discovery and engineering (NTRs, OYEs and allied oxidoreductases) pertinent to *p*-aminobenzenesulfonamide manufacture; (iii) highlight process intensification strategies such as whole-cell versus cell-free, immobilisation, continuous flow, electro-/photobiocatalysis for efficient cofactor management and product isolation; and (iv) discuss the quantitative green metrics and risk profiles. Through the combination of mechanistic, engineering and sustainability perspectives, we outline a practical roadmap for delivering sulfanilamide cores through enzyme-enabled pathways that meet contemporary performance and environmental benchmarks while opening new space for chemoselective transformations under benign conditions.

Although studies on nitroreductases and related biocatalytic platforms are available, direct experimental evidence specifically on nitro-sulfonamide substrates such as *p*-nitrobenzenesulfonamide (*p*-NBS) remains extremely limited. The above stated objectives of this review have been met in this review using three categories of information: (i) direct evidence obtained with nitro-sulfonamide substrates (currently limited), (ii) indirect evidence derived from structurally or electronically related nitroarenes that can reasonably be extrapolated, and (iii) speculative proposals for enzyme engineering, process integration, and scalability that rely on analogy and require targeted validation on the target substrate. This critical distinction is maintained to highlight the areas where dedicated substrate-specific studies are still urgently needed.

## 2 The core transformation: reduction of *p*-Nitrobenzenesulfonamide to *p*-Aminobenzenesulfonamide

**FIGURE 1:**
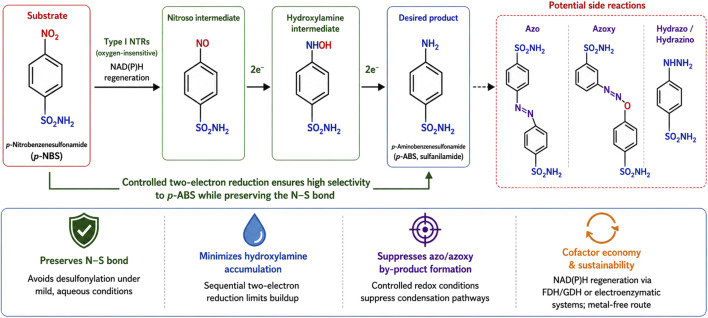
Integrative overview of the selective biocatalytic nitroreduction of *p*-nitrobenzenesulfonamide (*p*-NBS) to *p*-aminobenzenesulfonamide (*p*-ABS) mediated by Type I nitroreductases (NTRs). The reaction proceeds through nitroso and hydroxylamine intermediates with sequential two-electron reductions while minimizing side reactions (azo, azoxy, and hydrazo/hydrazino formation, and preserving the sulfonamide (N–S) bond.

We focus on the selective nitro reduction of nitro-sulfonamides to amino-sulfonamides, exemplified by converting *p*-nitrobenzenesulfonamide (*p*-NBS) to *p*-aminobenzenesulfonamide (*p*-ABS, sulfanilamide). This process, shown in Figure 1, requires reducing the nitro group (-NO_2_ to -NH_2_) while maintaining sulfonamide (-SO_2_NH_2_) integrity, which is susceptible to cleavage in harsh conditions. The figure also represents mild aqueous conditions of Type I nitroreductase catalysis together with the key chemoselectivity challenges and comparison with conventional routes.

The reaction shows benzene ring with para-substituted sulfonamide and nitro groups in the substrate, yielding *p*-ABS, a key intermediate for sulfonamide pharmaceuticals like antibiotics and enzyme inhibitors (Chhablani et al., 2025; Noor et al., 2025). Traditional methods (Pd/H_2_ or Béchamp) often compromise sustainability and selectivity, motivating biocatalytic alternatives using Type I nitroreductases (NTRs) under mild aqueous conditions to minimize N-S cleavage and byproduct formation (for example, hydroxylamine, azo/azoxy).

## 3 Chemical baseline: how nitro-sulfonamides are reduced today

### 3.1 Precious metal-based hydrogenation (state of practice)

Catalytic hydrogenation over supported precious metals such as Pd and Pt remains a well-established industrial approach for converting nitroaromatics to anilines, delivering strong activity. Recent reviews and studies dissect the canonical direct and condensation pathways (via nitroso/hydroxylamine → aniline, or via azo/azoxy coupling), which demonsrate typical impurity profiles (nitroso-, hydroxylamine-, azo-, azoxy-species) and the need for kinetic/selectivity control (Wu et al., 2024; Liu et al., 2025). In functionalised substrates, selectivity challenges become more complex; for example, hydrogenation of halogenated nitroarenes is a classic case where dehalogenation competes with nitro reduction. Therefore, it requires precise catalyst and condition control, the principles directly relevant to safeguarding other sensitive groups (Loos et al., 2016).

### 3.2 Sulfur tolerance and poisoning

A critical issue for Pd/C in multipurpose plants is sulfur-induced deactivation from S-bearing substrates, impurities, and sulfurous feeds. Sulfur binds strongly to metal sites, promoting deactivation and coke/azo deposits. This has resulted in development of sulfur-tolerant designs (for example, heteroatom-doped carbons, oxide-modified metals) and “antidote” strategies. Studies highlight defect-rich Pt/CeO_2_ catalysts with enhanced tolerance towards S-containing nitroarenes, sulfur-doped carbon-supported Pd with tuned electronic properties, and process data linking sulfur poisoning to increased azo deposits on Pd/C (Zhang et al., 2023; Ren et al., 2025; Zhang et al., 2013). Regeneration and metal recovery are critical strategies to address cost and sustainability challenges; practical protocols, such as a solvent/acid wash-regeneration using chloroform and glacial acetic acid have been developed to restore activity to spent Pd/C and Pd(OH)_2_/C catalysts. This achieves high yields comparable to fresh catalysts while palladium recovery from reaction streams is increasingly integrated and standardized in pharmaceutical processes to meet regulatory limits and reduce waste (Zhang Q. et al., 2022; Economidou et al., 2023).

Continuous-flow transfer hydrogenations using the CuNPs/Celite catalytic system have broadened safe operating windows by eliminating the need for hydrogen gas and improving heat and mass transfer. In a recent study, it was demonstrated that the system achieved > 99% conversion of nitrobenzene in endurance tests lasting up to 145 h. These advances are relevant for nitrobenzene reduction, with the protocol showing versatility across different nitrobenzene concentrations and hydrogen donors such as ethylene glycol and glycerol, as already reported (Martina et al., 2024). Precious-metal stewardship and impurity management remain persistent challenges in pharmaceutical manufacturing, primarily due to the high cost and limited supply of palladium, and the need to meet strict regulatory limits on residual metal impurities. These challenges are compounded by the increasing emphasis on sustainable practices and the economic pressure to recover and recycle palladium from process streams (Economidou et al., 2023).

### 3.3 Béchamp iron/acid reductions (legacy and “greener” variants)

The historic Fe/acid Béchamp route was widely used for nitroarene reductions but has been largely replaced by palladium-catalyzed hydrogenation, which produces purer products at lower cost. As a result, catalytic hydrogenation has become the preferred industrial route for amine production (Figueras and Coq, 2001). Current research is exploring the low cost iron while addressing waste, as seen in recent mechanochemical iron reductions that suppress bulk solvents and streamline separations. However, these approaches are still emerging and have not yet been widely industrialised (Bahadur et al., 2025).

### 3.4 Challenges of nitro-sulfonamides

Two considerations dominate:

#### 3.4.1 Electronic deactivation of the ring

Substituent effects can influence the rate of nitro reduction by iron, as shown by kinetic differences between nitrobenzene and methyl-substituted derivatives. While electron-donating groups appear to accelerate reduction, further studies would be required to map these trends onto more strongly deactivated substrates such as nitro-sulfonamides (Liu et al., 2025).

#### 3.4.2 Chemoselectivity versus N-S bond integrity

Desulfonylation has emerged as a credible reaction under both photoreductive and chemical reduction conditions. Radical and anionic desulfonylation pathways enabled by metals, electrolysis, and photoredox systems have been reported. More recent studies confirm that N-S bond cleavage is possible even under mild conditions, including metal-free photocatalysis and polysulfide-mediated platforms. This demonstrates the importance of chemoselectivity when sulfonamides serve as functional or protective groups (Pavlovska et al., 2023; Liu et al., 2025; Chu et al., 2021).

### 3.5 Selectivity target for *p*-aminobenzenesulfonamide

Selective reduction of para-nitro groups to amines is a key objective in pharmaceutical synthesis. The achievement of this transformation without side reactions such as dehalogenation or functional group loss is critical. Although Pd/H_2_ platforms can offer high selectivity when carefully modified; recent enzyme-enabled reductions under mild aqueous conditions have shown excellent chemoselectivity, preserving halogens and other labile groups. These biocatalytic systems offer promising alternatives for substrates bearing sensitive compounds, where traditional metal-catalyzed methods may cause selectivity challenges (Sokolova et al., 2024; Li Z et al., 2024; Yao et al., 2024).

Pd-catalysed hydrogenation remains the industrial standard for nitroarene reduction due to its efficiency and selectivity, though there are safety, cost, and functional group tolerance challenges. Traditional Béchamp (Fe/acid) routes offer a low-cost alternative but are environmentally not feasible due to high waste generation and complex processes. Selectivity remains a challenge when reducible functional groups are present. These challenges motivate scientists to explore milder, selective, and sustainable alternatives such as non-precious metal catalysts or biocatalytic platforms (Liu et al., 2025; Chu et al., 2021).

As the direct experimental datasets for the *p*-nitrobenzenesulfonamide (p-NBS) to *p*-aminobenzenesulfonamide (*p*-ABS) system remain limited, the comparative analyses presented in Tables 1–3 later in the manuscript integrate three evidence categories: (i) direct observations obtained with nitro-sulfonamide substrates where available, (ii) indirect evidence extrapolated from structurally or electronically related nitroarenes and nitroheterocycles, and (iii) forward-looking process and engineering proposals derived from established principles in biocatalysis and pharmaceutical process intensification. Accordingly, these tables are intended as evidence-mapped translational frameworks rather than definitive industrial validation datasets for the *p*-NBS/*p*-ABS system. Table 1 below presents the evidence structure for reduction pathways in nitro-sulfonamides.

**TABLE 1:** Comparative evidence framework for reduction routes relevant to nitro-sulfonamide aromatics.

Route/Catalyst	Typical conditions	Core strengths	Key liabilities for sulfonamides	Common impurity profile	Residuals/Stewardship	Green metrics and safety notes	Industrial maturity
Pd/C (H_2_ hydrogenation)	1–10 bar H_2_; RT-80 °C; polar protic/MeOH/EtOAc; modifiers possible	Fast, broadly applicable; robust tool-of-trade	S-poisoning risk; N-S cleavage/deprotection risks; metal residues	Hydroxylamine, azo/azoxy, dehalogenation side-products	Precious-metal residues; recovery/ICH Q3D compliance	Low E-factor possible but metal lifecycle + hydrogen handling overhead	High (industrial nitroarene benchmark; limited *p*-NBS-specific data)
Béchamp (Fe/acid)	Fe filings + acid; slurry; heat	Cheap reagents; tolerant	Large Fe-sludge; complex work-up; variable selectivity	Azo/azoxy; tars	Heavy solids waste	Poor PMI/E-factor; high downstream burden	Legacy/declining (well-established nitroarene process)
Transfer hydrogenation (CuNP/Celite, etc.)	Alcohol donors (EG/glycerol); flow; mild	H_2_-free; safer windows	Substrate-specific tuning; donor residues	Azo/azoxy if electron supply mis-matched	Non-precious residues	Better safety; solvent/donor PMI trade-offs	Emerging (substrate-dependent nitroarene evidence)
Type I NTR (NfsA/NfsB) + NAD(P)H	pH 7–7.5, 20 °C–30 °C; aqueous; ≤10% co-solvent	Air-tolerant two-electron steps; chemoselective; low-pressure	May stall at hydroxylamine; enzyme stability/solubility limits	Hydroxylamine build-up → azoxy/azo if uncontrolled	Proteinaceous residuals (filterable)	Excellent PMI potential; metal-lean; benign conditions	Pilot-scale potential; direct substrate-level evidence remains limited
Immobilised NTR packed-bed (with GDH/FDH)	Flow; co-immobilised recycling; PAT	Continuous; reuse; short RT; low hold-up	Cofactor cost (GDH) or CO_2_ handling (FDH)	As above, but controllable with PAT	Minimal metals	Good PMI; continuous QA via PAT	Conceptual-to-lab-flow translational framework
Electro-NAD(P)H (mediator cathodes)	PEM cells; set potential/current	Sacrificial-substrate-free; modular	Mediator selection; current density limits	Selectivity sensitive to potential	Trace mediator	Electric-to-chem value; easy skid scaling	Rapidly emerging electroenzymatic platform
[NiFe]-hydrogenase on carbon (cofactor-independent)	1 bar H_2_; aqueous; mild	Full 6-e^-^ to amines; precious-metal-free	System transferability to sulfonamides to be proven	Typically clean; monitor hydroxylamine	Enzyme/protein residuals	Excellent safety/PMI; H_2_ logistics minimal	Early-stage nitroarene evidence; sulfonamide transferability unproven

Industrial maturity assessments combine direct experimental evidence, nitroarene related literature, and mechanistic insights translated into process engineering proposals.

## 4 Biocatalytic nitro-reduction landscape

Flavin-dependent nitroreductases (NTRs) are the best-studied enzyme class for reducing aromatic nitro groups. Type I (oxygen-insensitive) NTRs perform two-electron transfers from reduced flavin (via NAD(P)H) to the nitro group, proceeding through nitroso and hydroxylamine intermediates and typically giving anilines. Type II (oxygen-sensitive) NTRs participate in single-electron transfers that can redox-cycle with O_2_. Recent studies emphasize the synthetic potential of NTRs, including broad activity across nitroaromatics and nitroheterocycles. Benchmark kinetic and mode of action studies with the *Enterobacter cloacae* and *E. coli* NfsA/NfsB families continue to anchor the field, mapping the nitroso → hydroxylamine → amine sequence and the cofactor-coupled ping-pong kinetics showing the way an enzyme can work on different substrates. Importantly, the latest studies also provide mechanistic insights (active-site polarity/sterics and cofactor-binding residues) for modifying activity and selectivity (Russo et al., 2025; Valiauga et al., 2018; Valiauga et al., 2024; Luján et al., 2024).

### 4.1 Breadth and engineering of NTRs

NTRs accommodate a wide electronic span of nitroarenes with several studies showing rate trends tracking Hammett σ (that is, deactivated/EWG substrates can still be competent, though often slower). Structure-function analyses and recent variant design efforts (that is, NfsB F70A/F108Y lineage) show that small changes in the enzyme’s structure can adjust the way cofactor and substrate fit together. Thus, making the enzyme work better on nitro compounds that are normally difficult to process. New stabilization strategies such as encapsulation of NfsB dimers inside protein nanocompartments are also emerging to raise operational stability under process-like conditions (Miller et al., 2018; Sharrock et al., 2024; Zmyslia et al., 2025).

### 4.2 Type I versus type II in practice

**FIGURE 2:**
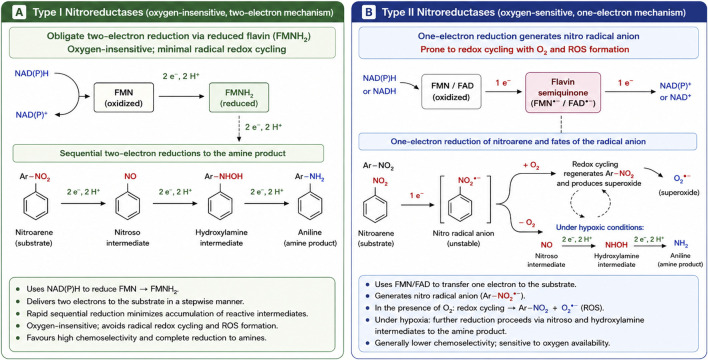
Comparative catalytic mechanisms of Type I and Type II nitroreductases.

In biocatalytic applications, oxygen-insensitive Type I nitroreductases are often preferred because they perform two electron reductions, avoid formation of semiquinone intermediates, and reduce the risk of unproductive redox cycling with molecular oxygen. In contrast, Type II NTRs catalyze one electron transfers that generate reactive nitro radical anions, which can react with oxygen to form superoxide and other reactive oxygen species. These mechanistic differences are important when selecting enzymes for use in redox sensitive or oxygen exposed environments (Čėnas et al., 2021). Type I and Type II nitroreductases differ fundamentally in electron-transfer mode and oxygen response. These contrasting mechanisms are summarized in Figure 2.

### 4.3 Old Yellow Enzymes (OYEs)

OYEs are FMN dependent ene-reductases best known for asymmetric C=C reductions; however, studies have shown they also engage nitro-electrophilic substrates under suitable mechanistic conditions. Other reports have highlighted OYE-catalysed nitro-olefin reductions proceeding through hydride addition followed by enzyme-mediated aci-nitro tautomerization. More recent mechanistic and structural studies have revealed hydride transfers to non-traditional acceptors such as nitro groups, indicating an expanding catalytic repertoire and potential for engineering to carry out different functions. However, their activity on nitro substrates is substrate dependent, most effective with nitro-olefins and not broadly general across nitroarene classes (Sahrawat et al., 2024; Meah and Massey, 2000).

### 4.4 Auxiliary reductive platforms that pair well with enzymes

Two directions are particularly relevant for greener cofactor economy and chemoselectivity:

#### 4.4.1 Hydrogenase powered electrobiocatalysis

A recently demonstrated platform channels electrons from H_2_ (through a [NiFe]-hydrogenase wired to carbon black) directly to reduce over 30 diverse nitroarenes in water at 1 bar H_2_, delivering high yields and excellent functional-group tolerance. The system operates under mild, precious-metal-free conditions, and offers long catalyst lifetimes establishing a stand-alone paradigm for sustainable, biocatalytic hydrogenation (Sokolova et al., 2024).

#### 4.4.2 Photoenzymatic reduction strategies

Complementary photo-biocatalytic approaches can create reducing equivalents *in situ* and steer radical intermediates. This broadens the reaction envelope for difficult nitro substrates and related electrophiles while reducing reliance on expensive nicotinamides. Recent work illustrates the way modified photoenzymatic systems modulate intermediate lifetimes to favor productive reduction pathways (Luján et al., 2023).

Although the cited studies provide robust indirect evidence for the mechanistic versatility of Type I NTRs, OYEs, and auxiliary platforms on a broad range of nitroarenes, direct data on nitro-sulfonamide substrates are absent. Most conclusions in this section are therefore based on extrapolation from simpler or electronically analogous nitro compounds. While the shared two-electron reduction mechanism supports reasonable confidence in applicability, the strongly electron-withdrawing sulfonamide group may influence binding affinity, hydroxylamine accumulation, and N-S bond stability in ways that have not yet been experimentally verified.

### 4.5 Process relevant implications

These advances show a toolkit for aqueous, low-pressure, metal-lean nitro reductions using non-noble metals and alternative electron donors. This includes: (i) metal-based reductions (for example, Zn or Fe) under ultrasound, microwave, or CO_2_-assisted conditions; (ii) borohydride, hydrazine, or alcohol-mediated catalytic systems modified for selectivity toward hydroxylamines; and (iii) electrochemical and photochemical platforms for reducing nitroaromatics using *in situ* generated active hydrogen species. In these methods, the electron-supply strategy whether via H_2_, hydrazine, electrolysis, or light has emerged as a critical design along with catalyst surface modification. This has direct implications for impurity profiles (nitroso, hydroxylamine versus, azo, azoxy), solvent optimization, and scalability (Yu et al., 2024). Table 2 provides a summary of biocatalytic reduction lansdscape:

**TABLE 2:** Evidence-informed nitroreductase engineering and process platforms relevant to nitro-sulfonamide reduction.

Enzyme class	Electron transfer and O_2_ behavior	What it’s best at	Limiters for *p*-NBS/*p*-ABS path	Proven/Indicative engineering levers	Immobilisation and format	Best-fit cofactor supply
Type I NTR (NfsA/NfsB lineages)	Two-electron hydride equivalents; O_2_-insensitive	Mechanistically favored nitroso→hydroxylamine→amine progression under aerobic conditions	Endpoint to aniline can stall if redox not matched	NfsB F70A/F108Y access-tunnel tuning; active-site polarity; cofactor-binding tweaks	Covalent carriers and co-immobilised packed-bed configurations proposed from related nitroarene systems	FDH for atom economy; GDH for simplicity; Electro-NAD(P)H for continuous
Type II NTR	One-electron; O_2_-sensitive (radical anion cycles)	Hypoxic or oxygen-controlled reduction environments	Needs deoxygenation; ROS risk	Sequence mining; anaerobic process design	Possible but adds O_2_ control burden	Electro-NAD(P)H under inert conditions
OYEs (ene-reductases) – adjunct	FMN hydride chemistry; substrate-dependent nitro engagement	Mechanistically complementary and photo-assisted reduction cases	Not general for nitroarenes; case-by-case	Photochemical mode switching; active-site repurposing	Free enzyme or immobilised adjunct	As per paired system

**TABLE 3:** Hypothesis-driven process-integration and scalability framework for p-aminobenzenesulfonamide (p-ABS) biocatalysis.

Unit operation/Module	Preferred setting for first-pass pilot	Illustrative starting benchmarks (hypothesis-driven)	PAT/Control signals	Risk addressed	Scale-up note
Bioreactor: immobilised NTR packed-bed	Covalent carrier; 20 °C–30 °C; pH 7.0–7.5; water-rich (≤10% co-solvent)	Conceptual residence-time window (τ ≈ 5–15 min); superficial velocity to keep Δp low; ionic strength moderate	Online IR (NO/NOH bands), at-/online LC (substrate/amine/azoxy), Δp and RTD	Hydroxylamine build-up; azo/azoxy drift; mass-transfer limits	Number-up parallel beds; simple thermal control
Cofactor regeneration A (FDH loop)	FDH + NADP^+^; Na formate feed	Initial formate excess proposed from related FDH-driven systems; pH stat; CO_2_ stripping	pH, DO (informative), LC formate/formate drift	By-product minimisation, pH stability	Excellent atom economy; easy to PLC
Cofactor regeneration B (Electro-NAD(P)H)	PEM cell; fixed potential; iridium-polymer/mediator	Cathode potential/current density per mediator; recirculation	Cell voltage; FE; LC NAD(P)H/NAD(P)^+^	Substrate-free electrons; mediator carry-over	Modular skids; plug-and-play
Membrane phase splitter/ LLE	Zaiput-type; EtOAc or greener alt	Phase ratio 1:1–1:2; T 20 °C–25 °C	Flow stability; outlet composition	Fast removal of amine; suppress over-reduction	Straightforward scale-out
Polishing and crystallisation	Anti-solvent or cooling from aqueous mother liquor	Seeded; supersaturation control	In-process turbidity/ATR-IR; qNMR for API	Purity, particle control	Potentially transferable downstream framework
Green metrics and LCA gate	PMI/E-factor dashboard + gate-to-gate LCA vs. Pd/H_2_	Illustrative sustainability targets proposed from green pharmaceutical manufacturing benchmarks	Run-to-run PMI; energy kWh/kg; LCA hot-spots	Burden shifting	Comparative framework for future process-selection studies

The engineering strategies and process configurations summarized in this table combine direct mechanistic observations from nitroreductase literature with extrapolated insights from structurally related nitroarene systems. Studies on validation for *p*-nitrobenzenesulfonamide (*p*-NBS) substrates remains limited for several emerging immobilization and process-integration platforms.

The representative nitroreductases and Old Yellow Enzymes (OYEs) reported for nitro-group reduction and discussed throughout this review are summarized together with source organisms, UniProt accession information (where available), and corresponding literature references in Supplementary Table S1.

**FIGURE 3:**
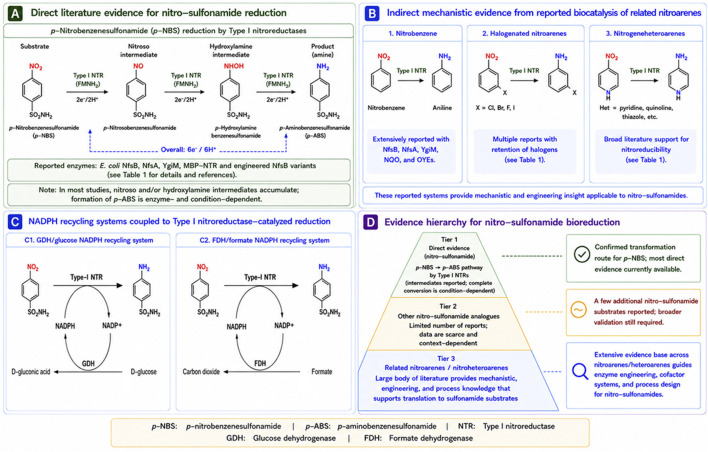
Evidence base and cofactor regeneration strategies for biocatalytic reduction of *p*-nitrobenzenesulfonamide (*p*-NBS) to *p*-aminobenzenesulfonamide (*p*-ABS) as adapted conceptually from Maier et al., 2024 (Ma et al., 2024).

As mentioned above the direct reports involving nitro-sulfonamide substrates remain comparatively limited, a substantial body of nitroreductase literature provides mechanistic, enzymatic, and process-engineering insights relevant to *p*-nitrobenzenesulfonamide (*p*-NBS) reduction. Figure 3 summarizes the current evidence base by distinguishing direct nitro-sulfonamide studies from broader nitroarene biocatalysis literature and the associated cofactor-regeneration strategies commonly employed in FMN-dependent nitroreductase systems.

As illustrated in Figure 3, direct experimental evidence is currently strongest for a limited number of nitro-sulfonamide substrates, whereas many enzyme-engineering, cofactor-regeneration, and process-integration concepts are supported by work on related nitroarene and nitroheteroarene systems. This distinction is important when assessing the current maturity of proposed nitro-sulfonamide biocatalytic platforms.

## 5 From nitro-sulfonamide to *p*-aminobenzenesulfonamide: substrate/mechanistic considerations

### 5.1 Electronic/steric features and enzyme choice

*P*-Nitrobenzenesulfonamide (*p*-NBS) contains both a para-nitro group and a sulfonamide moiety which are strongly electron-withdrawing and expected to reduce the electron density of the aromatic π-system. For flavin-dependent nitroreductases (NTRs), such electron-deficient substrates are energetically feasible. Multiple mechanistic and structure-dactivity studies have shown that nitroaromatic reduction rates often correlate positively with Hammett σ values, that is, more electron-withdrawing substituents can enhance the initial reduction steps. The canonical two-electron pathway typically proceeds through sequential formation of the nitroso and hydroxylamine intermediates. However, complete reduction to the aniline is conditional and depends on both enzyme architecture and redox potential; not all NTRs catalyze this final step efficiently. Enzymes capable of producing aromatic amines often accommodate larger π-systems and provide favorable active-site environments, sometimes supported by unique dimeric stabilization or optimized proton transfer capability (Miller et al., 2018; Čėnas et al., 2021).

Recent structural and mechanistic work on *E. coli* NfsA and engineered NfsB variants show that NTRs catalyze discrete two-electron (hydride) transfers from reduced flavin to the nitro group, without accumulation of flavin semiquinone intermediates. In NfsB, active site remodelling such as the F70A/F108Y substitutions enhances activity on demanding substrates like SN33623, CB1954, and metronidazole by widening substrate access channels and repositioning key residues. Computational modelling suggests that these changes may also favor substrate orientations conducive to hydride transfer (Valiauga et al., 2024; Sharrock et al., 2024).

By contrast, Old Yellow Enzymes (OYEs; ene-reductases) are not native nitro-group reductases for aromatic substrates; their classical role lies in the asymmetric reduction of activated C=C bonds in enones. Although recent developments in photoenzymatic catalysis have enabled OYEs to engage nitro substrates under photochemical conditions. This reactivity has been demonstrated primarily for aliphatic nitro compounds and select ketone reductions, proceeding through nitroso and hydroxylamine intermediates. A recent study reported dedicated nitroreductases (for example, BaNTR1 and EcNR) under light-driven conditions to achieve efficient and selective reductions of both aliphatic and aromatic nitro compounds. This provided broader applicability than previously reported OYE-based systems (Luján et al., 2023).

### 5.2 Hydrazino-sulfonamide formation and deep reduction pathways

The canonical two-electron reduction sequence of aromatic nitro groups (nitro, nitroso, hydroxylamine, aniline) has been extensively documented in both chemocatalytic and enzymatic systems (Guo et al., 2025; Čėnas et al., 2021). The nitroaromatic substrates may undergo alternative or deeper reduction pathways under strongly reducing, radical, or electrochemical conditions. The mechanistic analysis of nitroarene reductions show that intermediate nitrogen species can participate in off-pathway reactions, such as N-N coupling and over-reduction, depending on the electron flux and reaction environment (Chu et al., 2021; Chong et al., 2020). Therefore, hydrazino-substituted aromatics represent a theoretical deep-reduction or rearrangement product class, structurally characterized by the replacement of the amino group with a hydrazino fragment (-NH-NH_2_) at the same time preserving the sulfonamide moiety.

Mechanistically, the formation of abnormal nitrogen-nitrogen bond formation is predominantly associated with radical mediated or single-electron transfer pathways. Studies related to nitroaromatic reduction chemistry describe the way nitroso and hydroxylamine intermediates can participate in condensation reactions, forming azoxy, azo, and related N-N coupled species when reduction conditions are not tightly regulated (Guo et al., 2025; Chu et al., 2021). Furthermore, the electrochemical studies highlight that modulation of reduction potential can shift selectivity among azoxy-, azo-, and fully reduced amine products. This demonstrates the sensitivity of nitrogen speciation to electron delivery rates (Chong et al., 2020).

More importantly, the likelihood of azoxy or N-N linked condensation product formation is significantly lower under controlled two-electron enzymatic systems with non-enzymatic reducers like ascorbate. Type I (oxygen-insensitive) nitroreductases catalyze the obligate two-electron hydride transfers from reduced flavin without accumulation of semiquinone intermediates; therefore, minimizing radical pathways associated with single-electron reductions (Valiauga et al., 2018; Čėnas et al., 2021). This mechanistic constraint constitutes a major chemoselectivity advantage of enzymatic nitroreduction over classical metal or photoredox-driven systems.

However, under mismatched redox supply, incomplete turnover, or hybrid chemoenzymatic regimes, the build-up of nitroso and hydroxylamine intermediates can still promote off-pathway reactions, such as N-N condensation or further transformation. Accordingly, tight control of electron supply, residence time, and real-time analytical monitoring (LC/IR PAT) is also essential to ensure selective conversion to *p*-aminobenzenesulfonamide while suppressing deeper or alternative nitrogen reduction pathways (Cosgrove et al., 2023; Simon et al., 2022). The formation of a hydrazino derivative remains a theoretical possibility rather than a commonly observed product in standard Type I NTR catalysis.

The primary mechanistic challenges for the biocatalytic reduction of *p*-nitrobenzenesulfonamide (*p*-NBS) to *p*-aminobenzenesulfonamide (*p*-ABS) using Type I nitroreductases are the accumulation of the hydroxylamine intermediate and its subsequent non-enzymatic condensation with the nitroso species to form azoxy and azo by-products. For *p*-NBS, typical nitroreductases (and many engineered variants) predominantly yield the hydroxylamine with little to no formation of the desired amine. In broader nitroarene reductions, accumulation of nitroso and hydroxylamine intermediates readily leads to spontaneous non-enzymatic condensation into azoxy (and further azo) products, especially when conditions favor their build-up. As a result, tight control of the redox environment and electron supply is essential to promote complete reduction to the desired aniline while suppressing off-pathway nitrogen species (Park et al., 2015; Luján et al., 2023).

### 5.3 Selectivity risks (hydroxylamine build-up and over-reduction)

In chemical and (photo) biocatalytic nitro reductions; the accumulation of N-hydroxylamine can cause side reactions. Condensation of nitroso with hydroxylamine gives azoxy species, that can cascade to azo/hydrazo by-products. Therefore, careful electron-supply and medium control are essential to drive complete conversion to aniline (Guo et al., 2025; Simon et al., 2022; Wumaer et al., 2022).

Type I (oxygen-insensitive) NTRs are generally preferred for preparative nitro reductions because they perform net two-electron steps in air without generating nitro radical anions that redox-cycle with O_2_ (Roldán et al., 2008; Verma et al., 2024).

Incomplete reductions have been observed in practice, as nitroreductases (NTRs) often stall at the hydroxylamine stage. However, process designs that pair NTRs with complementary catalysts or intensified formats can significantly improve endpoint selectivity in many cases. For example, continuous packed-bed NTR systems and synergistic NTR/vanadium cascades have achieved high conversions. Moreover, for a broad range of nitroarenes, have yielded predominantly clean aniline products though selectivity may vary depending on substrate structure (Bisagni et al., 2022; Cosgrove et al., 2023).

### 5.4 Medium engineering: pH, ionic strength, and cosolvents (including green media)

NTRs such as *E. coli* NfsA exhibit optimal turnover under near-neutral, buffered aqueous conditions (pH 7.0), consistent with the flavin redox thermodynamics as confirmed by potentiometric studies (E_0_’ = −215 mV). Although the influence of varying ionic strength was assessed using substrates of different charge types, results showed minimal perturbation to catalytic efficiency across the tested range. Mechanistic analyses such as activation entropy and enthalpy studies, as well as molecular modelling, revealed that fine-tuning of redox states and hydride transfer steps is indeed sensitive to the local microenvironment within the enzyme’s active site, particularly through short-range interactions such as hydrogen bonding with key residues (for example, Arg15, Ser40, Arg133) (Valiauga et al., 2024).

In case of increasing substrate solubility limits which are commonly observed with many industrially relevant apolar compounds, small fractions of water-miscible cosolvents, such as DMSO or ethanol, are frequently used in biocatalysis to enhance solubility. However, these must be used with caution, as even low concentrations can significantly reduce enzyme activity or stability. Studies on enzyme behavior in cosolvent systems emphasize the importance of maintaining “water-rich” conditions to retain biocatalyst performance (Schie et al., 2021).

From a green-chemistry perspective, deep eutectic solvents (DES) and bio-based eutectic mixtures are increasingly explored as sustainable alternatives to traditional organic solvents for reduction reactions. In a recent study, such DES systems particularly choline chloride-based mixtures were effectively employed as the primary reaction medium for the chemoselective reduction of nitroalkenes using ammonia borane, a mild and atom-economical reducing agent. A variety of substituted nitroalkenes were reduced to the corresponding nitroalkanes with fair to good yields, and the protocols demonstrated solvent recyclability and avoided the use of conventional organic solvents in product isolation. These findings showed the viability of bio-derived DES in green chemical reductions under purely chemocatalytic conditions (Faverio et al., 2021).

### 5.5 O_2_ management and enzyme class

The oxygen sensitivity of nitroreductases (NTRs) directly maps onto their classification into Type I and Type II. Type I enzymes, including the NfsA and NfsB families, are oxygen-insensitive and can operate effectively in the presence of molecular oxygen. These enzymes catalyze two-electron reductions of nitroaromatic compounds to produce amino derivatives via nitroso and hydroxylamine intermediates. Their oxygen-insensitivity makes them functionally distinct from Type II enzymes, which are oxygen-sensitive and perform one-electron reductions under hypoxic conditions (Verma et al., 2024).

Type II (oxygen-sensitive) NTRs catalyze one-electron reductions that generate nitro radical anions prone to ineffective O_2_ cycling. These require deoxygenation or strict anaerobiosis to achieve productive turnover (Roldán et al., 2008).

In case of nitroaromatic prodrugs such as SN33623, metronidazole, and CB1954, a Type I nitroreductase like NfsB is a pragmatic first choice due to its oxygen-insensitive catalytic mechanism. When baseline rates are modest, engineered NfsB variants such as the F70A/F108Y double mutant provide additional support for enhancement through altered active-site dynamics, including widened substrate access channels and favorable hydrogen bonding interactions (Sharrock et al., 2024).

### 5.6 Analytical control of intermediates and product quality

The reaction intermediates such as nitroso and hydroxylamine species are known to influence product selectivity in nitroarene reductions. Therefore, it is important to implement in-process analytical strategies capable of tracking their impact. As the direct, routine quantification of these intermediates remains analytically challenging, product-based signatures particularly the formation of azoxy and azo species can offer indirect evidence for the presence and behaviour of nitroso and hydroxylamine intermediates. In the automated flow hydrogenation setup described by Simon et al., 2022 (Simon et al., 2022), inline FT-IR combined with atline UHPLC enabled high-frequency quantification of substrates, products, and by-products, including intermediate peaks suspected to correspond to hydroxylamine derivatives. Although LC-MS and GC-MS analysis did not conclusively identify these species, their indirect detection through reaction profiles and intermediate build-up was critical for process understanding. Similarly, in the electrosynthetic study by Chong et al., 2020 (Chong et al., 2020), careful potential tuning was shown to shift product selectivity among azoxy-, azo-, and aniline products, indicating modulation of intermediate lifetimes. These orthogonal strategies, real-time analytics and electrochemical control, together highlight the way data-rich approaches can inform intermediate suppression and optimize product selectivity in nitro reduction pathways.

In relation to the target amine, *p*-aminobenzenesulfonamide (*p*-ABS), a validated reversed-phase HPLC method with UV and PDA detection has been developed and successfully applied for its quantitative determination and impurity profiling in sulfonamide hydrochloride formulations. Additionally, robust HPLC-UV and HPLC-FLD methods have been reported for the analysis of structurally related sulfonamides in complex matrices such as animal-derived food products and organic fertilizers. Furthermore, UHPLC-MS/MS protocols have been validated for the simultaneous determination of multiple sulfonamides in challenging matrices like processed food, demonstrating high sensitivity, selectivity, and recovery. These methods, offer a strong foundation for adaptation to such samples following appropriate validation (Osiński et al., 2022; Aher et al., 2023; Iram et al., 2024; Li et al., 2023).

When combined with targeted monitoring of azoxy/azo intermediates using integrated FT-IR and UHPLC analytics, this automated toolbox enables robust real-time tracking of reaction outcomes and impurity profiles, facilitating precise control over endpoint composition in heterogeneous catalytic hydrogenations (Simon et al., 2022).

### 5.7 Evidence base and extrapolation to nitro-sulfonamides

Both batch and continuous nitroreductase (NTR) platforms have shown the clean conversion of diverse nitroaromatics to their corresponding anilines with examples such as 2-chloro-5-nitropyridine studies specifically involving sulfonamide linked nitroaromatics are limited. Therefore, current applications are supported by mechanistic understanding and empirical success with structurally related nitroarene substrates, along with general insights into NTR catalytic behavior from studies of enzyme engineering, substrate scope, and co-catalyst-assisted reductions (Russo et al., 2025; Miller et al., 2018; Cosgrove et al., 2023).

This gap highlights the value of a short feasibility screen, that is, (i) Type I NTR panel (wild-type and engineered NfsB); (ii) water-rich media with low cosolvent; (iii) O_2_-tolerant operation. If needed, process intensification using immobilized flow or chemoenzymatic coupling can be utilized to secure full conversion and high purity (Bisagni et al., 2022; Cosgrove et al., 2023).

## 6 Enzyme discovery & engineering

**FIGURE 4:**
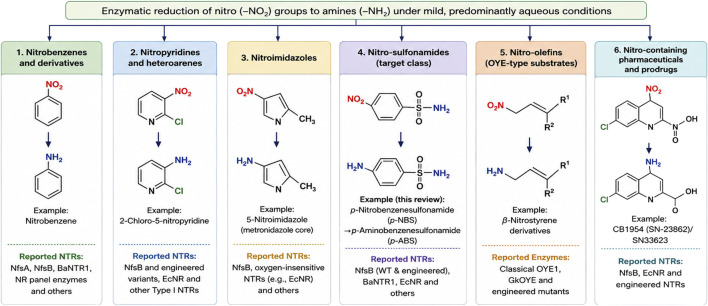
Representative substrate classes reported for nitroreductase- and flavin-dependent reductase-catalyzed reduction.

Beyond *p*-nitrobenzenesulfonamide, nitroreductases and related flavin-dependent reductases have been reported to transform a broad range of nitroaromatic, nitroheteroaromatic, pharmaceutical, and nitro-olefin substrates. Representative substrate classes relevant to the current biocatalytic literature are summarized in Figure 4.

The breadth of this substrate scope highlights the potential of nitroreductases as versatile biocatalysts and provides a foundation for extending selective nitro reduction to additional nitro-sulfonamide derivatives.

### 6.1 Landscape and discovery; mining the NTR superfamily with modern tools

**FIGURE 5:**
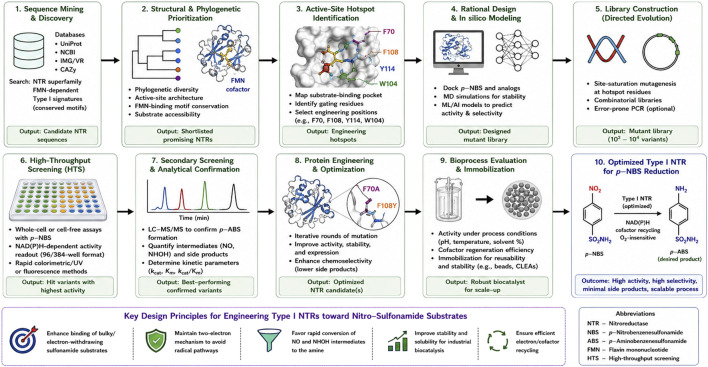
Enzyme discovery and engineering strategy map for developing Type I nitroreductases (NTRs) for the reduction of *p*-nitrobenzenesulfonamide (*p*-NBS) to *p*-aminobenzesulfonamide (*p*-ABS).

Nitroreductases (NTRs) form a large, functionally diverse FMN-dependent superfamily scattered across bacteria and some eukaryotes. Sequence similarity network (SSN) analysis have shown that the superfamily occupies broad, still underexplored sequence, function space, enabling rapid prioritization of clades that diverge in active-site chemistry for screening on new substrates (Copp et al., 2018). Modern nitroreductase engineering combines sequence mining, structure-guided design, directed evolution, and high-throughput screening to develop robust Type I NTRs with improved activity, chemoselectivity, and process compatibility for nitro-sulfonamide reduction (Figure 5).

Such integrated engineering workflows are expected to accelerate development of scalable, oxygen-tolerant biocatalysts capable of selectively converting challenging nitro-sulfonamide substrates under mild aqueous conditions.

A high expression metagenomic cloning system, incorporating optimized promoters, ribosome binding sites, and start codon positioning, enabled efficient functional screening of environmental DNA. Using this approach, the authors identified and validated 21 nitroreductases from diverse enzyme families including variants missed by standard metagenomic libraries. This highlighs that low gene expression in heterologous hosts can be a key limitation in functional metagenomics. It has been demonstrated that enhancing expression context, rather than focusing solely on sequence diversity, can substantially improve the recovery of functional enzymes from complex metagenomic samples (Rich et al., 2023).

Metagenome-assembled genome (MAG) studies are expanding our understanding of microbial diversity and metabolic potential in extreme and saline environments. In the Buhera soda pans of Zimbabwe, MAGs recovered from metagenomic data revealed a dominance of haloalkaliphilic taxa and included organisms that had not been previously cultured. Functional profiling of these MAGs identified diverse metabolic pathways, including those involved in carbohydrate metabolism, and key steps in the nitrogen and sulphur cycles. These findings demonstrate the value of MAGs in revealing the ecological roles and biotechnological potential of uncultured extremophiles in saline-alkaline systems (Mangoma et al., 2024).

Finally, biochemical genetics continues to reveal noncanonical roles (for example, lipid biosynthesis in *Mycobacterium tuberculosis*), highlighting the way evolution has repurposed the NTR scaffold and shown that mining should not be limited to “detoxification” annotations alone (Grigg et al., 2023).

#### 6.1.1 AI-assisted sequence function prediction

Machine learning (ML) models are increasingly applied to enzyme engineering, using supervised and semi-supervised learning trained on limited activity labels and structural features. These models have enriched functional variants and enabled beneficial mutations, with successful applications to improving enzyme activity, stability, and solubility (Kouba et al., 2023).

#### 6.1.2 Active-site hotspots; lessons from engineered NfsB

Sequence-activity analysis of phylogenetically related NfsB enzymes identified access channel and active-site residues as key determinants of substrate structure and catalytic rate. The double substitution F70A/F108Y markedly enhanced turnover of the 5-nitroimidazole PET probe SN33623. A high resolution crystal structure (1.98 Å) of the mutant enzyme revealed an expanded substrate access channel and new hydrogen bonding interactions that stabilize the active site environment. The follow-up computational modelling confirmed the improved activation profile and mapped the effects of these mutations on substrate specificity across the nitroaromatic compounds SN33623, CB1954, and metronidazole (Sharrock et al., 2024; Sharrock et al., 2023).

The representative engineered nitroreductase (NTR) and Old Yellow Enzyme (OYE) variants reported to exhibit enhanced catalytic or operational properties are summarized in Supplementary Table S2.

#### 6.1.3 Directed evolution workflows and high-throughput screening

State of the art directed evolution pipelines combine genetically diversified libraries, generated through random and semi-rational mutagenesis, with high-throughput screening methods such as microtiter plate-based assays. Library generation typically targets key residues in the substrate access tunnel and active site (for example, positions analogous to F70/F108 in NfsB). Validated assay formats include; (i) direct LC-MS analytics for quantifying substrate consumption or peptide degradation with minimal sample preparation in biological media, and (ii) fluorogenic probes such as resorufin based reporters that respond to nitroreductase activity and enable fluorescence based readouts compatible with phenotypic assays. Additional strategies include GFP-based reporter systems linked to SOS response for FACS enrichment and positive genetic selection using nitroimidazole prodrugs. Recent advancements demonstrate that streamlined LC-MS workflows can quantify dozens of analytes in complex cell culture environments efficiently and reproducibly (Xiao et al., 2015; Rozans et al., 2024; Tran et al., 2024). Multiple level workflows (fluorescence pre-screen followed by LC-MS validation on *p*-NBS analogs) combined with ML-guided prioritization accelerate convergence on variants with improved activity, chemoselectivity, and reduced hydroxylamine accumulation (Kouba et al., 2023).

### 6.2 Cofactor economy for scalable biocatalysis

Cofactor economy is a critical enabler for scalable biocatalysis. Enzymatic NAD(P)H-recycling systems such as glucose dehydrogenase (GDH) and formate dehydrogenase (FDH) are widely used due to their operational simplicity, cost-effectiveness, and compatibility with aqueous media. GDHs are well established for cofactor regeneration and are increasingly recognized for their multifunctionality in small-molecule synthesis such as ketone reduction and imine reduction (Ma et al., 2024; Kumar et al., 2025).

Electroenzymatic regeneration is increasingly attractive due to its advantages. Modern mediator-assisted cathodic systems such as those using iridium complexes, achieve high faradaic efficiency (up to 99%) and improved 1,4-NAD(P)H selectivity (91:9 M ratio of 1,4-NADH to 1,6-NADH), as reported in recent studies. These systems achieve reduction by utilizing electrical energy and water hence eliminating the need for sacrificial substrates. This produces only benign O_2_ which simplifies downstream purification by avoiding byproduct formation. Recent studies detail the mechanisms of NAD(P)H regeneration, categorize electron mediators and describe reactor designs, providing a foundation for efficient cofactor regeneration coupled with enzymatic catalysis (Li Y et al., 2024; Trotta et al., 2024).

The integration of H_2_-driven enzyme modules such as systems coupling hydrogenases with formate dehydrogenases shows a promising hybrid biocatalytic approach for CO_2_ reduction. These systems offer advantages in contexts where minimizing cofactor cost and byproduct formation is critical to process efficiency. It is because they enable high yield, atom efficient transformations using molecular hydrogen as a clean and inexpensive electron donor (Groh et al., 2024).

In practice, glucose dehydrogenase (GDH) and formate dehydrogenase (FDH) remain the common enzymatic systems for NAD(P)H regeneration. GDH is frequently applied due to its broad availability and compatibility with NAD^+^, although its use leads to gluconic acid accumulation which necessitates the pH control. In contrast, FDH offers significant advantages when minimizing by-product formation and maintaining pH stability is critical, since it converts formate cleanly to CO_2_. For process configurations involving continuous flow or multi-enzyme cascade systems, electrochemical NAD(P)H regeneration presents a compelling alternative. It eliminates the need for stoichiometric co-substrates, reduces by-product accumulation, and integrates well into modular bioreactors designed for efficient cofactor recycling (Ma et al., 2024; Li Y et al., 2024).

### 6.3 Aryl nitro-sulfonamides

A research approach would (i) mine SSN-defined NTR clades and metagenomes with expression-optimized capture to expand initial diversity; (ii) seed libraries around *nfsB* hotspots (for example, positions analogous to F70/F108) while sampling tunnel-lining and charge-network residues; (iii) apply dual-stage HTS-rapid probe/colorimetric triage followed by LC-MS quantification on aryl nitro-sulfonamides to suppress false positives; and (iv) pair hits with a cofactor recycling module chosen for process constraints (GDH for simplicity, FDH for atom economy, or electroenzymatic for minimal waste/continuous operations). As currently the ML models are ranking natural and designed variants between rounds, these steps collectively facilitate the discovery of NTRs modified for converting nitro-sulfonamides toward clean aniline endpoints (Copp et al., 2018; Rich et al., 2023; Sharrock et al., 2023; Rozans et al., 2024; Li Y et al., 2024).

Modern techniques for enzyme discovery and engineering have greatly expanded the nitroreductase toolbox. However, virtually all published screening and engineering efforts have been performed on model nitroarenes or prodrug substrates. Direct engineering or kinetic data on nitro-sulfonamides are lacking. The research approach outlined here therefore represents a high-priority speculative pathway that must be validated experimentally on *p*-NBS before claims of improved activity or selectivity can be made for the target transformation.

## 7 Process integration, scale-up, and sustainability for enzymatic nitro-sulfonamide reduction

**FIGURE 6:**
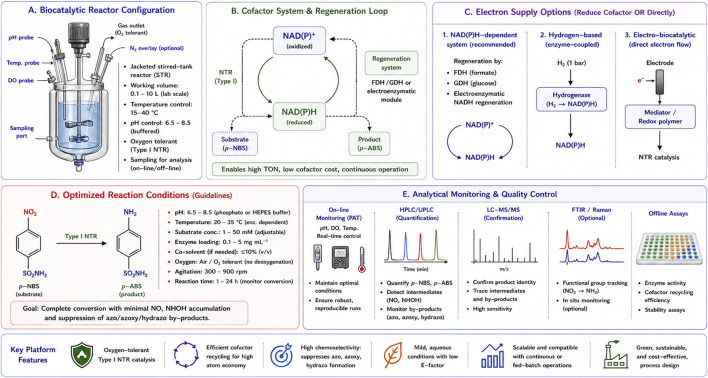
Conceptual process intensified biocatalytic framework for the reduction of *p*-nitrobenzenesulfonamide (*p*-NBS) to *p*-aminobenzesulfonamide (*p*-ABS). The platform integrates an oxygen-tolerant Type I NTR catalyst with efficient cofactor regeneration (FDH, GDH, or electroenzymatic systems), optimized reaction parameters, real time analytical monitoring, and scalable reactor configuration to achieve high conversion, excellent chemoselectivity, and minimal side product formation under mild, aqueous conditions.

Immobilization of oxidoreductases enhances their suitability for industrial biocatalysis by improving stability, enabling reuse, and simplifying separation, as has been reported. Two complementary immobilization strategies have been reported: carrier-bound methods, including adsorption, covalent attachment, and entrapment/encapsulation, and carrier-free approaches such as cross-linked enzyme aggregates (CLEAs). Each method presents adjustments in mass transfer, mechanical strength, and leaching risk. Covalent carrier-bound methods and CLEAs are noted for their enhanced operational stability, particularly when optimized. However, CLEAs show superior thermal stability and reusability, as reported in examples like genipin-cross-linked laccase CLEAs, while covalent bonding improves stability by reducing leakage (Sheldon, 2011; Robescu and Bavaro, 2025; Tadesse and Liu, 2025). Integration of oxygen-tolerant Type I nitroreductases with cofactor recycling, process analytical technologies, immobilization strategies, and scalable reactor configurations provides a conceptual framework for the future development of sustainable nitro-sulfonamide biocatalysis (Figure 6). Although substrate-specific process data for the *p*-NBS/*p*-ABS system remain limited, advances in continuous biocatalysis, cofactor regeneration engineering, immobilized enzyme systems, and electroenzymatic processing suggest that such integrated platforms may ultimately support scalable and chemoselective nitro-sulfonamide reduction under mild aqueous conditions.

Such process-intensified biocatalytic platforms are expected to improve conversion efficiency, reduce side-product formation, and facilitate translation of selective nitroreduction systems leading towards scalable pharmaceutical manufacturing.

Importantly for nitroreductases, a continuous packed-bed with an immobilized NTR (NR-55) has already been demonstrated to reduce aromatic nitro groups efficiently, establishing a precedent for steady-state operation, low hold-up, and facile scale-out by numbering-up (Cosgrove et al., 2023).

A recent study on flavin-dependent nitroreductases shows immobilized NTRs converting chloronitrobenzene to chloroaniline at lab scale with a reported yield of up to 60.9% in a 5 mL reaction volume. This highlights their potential as biocatalysts for sustainable synthesis of nitrogen-containing compounds (Russo et al., 2025).

### 7.1 Choosing a flow architecture

The continuous chemoenzymatic reduction of nitroaromatics, such as 2-chloro-5-nitropyridine, using the oxygen-insensitive Type I nitroreductase NR-55 in a packed-bed flow reactor achieves full conversion with residence times of 5–10 min and minimal azoxy dimerization (≤5%), facilitated by V2O5 as a co-catalyst for hydroxylamine reduction. Efficient NADPH recycling is enabled by co-immobilized glucose dehydrogenase GDH-101, utilizing 4 equivalents of glucose relative to substrate concentration. The immobilized system on amino-functionalized ECR8309F resin operates at 35 °C and atmospheric pressure in aqueous buffer. This shows reusability over multiple days with specific productivities up to 5.88 g gNR-55^–1^ for the model substrate and integrates with in-line Zaiput membrane separation using ethyl acetate for continuous product extraction. The system achieved isolated yields around 50%–58% (Cosgrove et al., 2023).

Electrochemical bioreactors with separate anode and cathode chambers, utilizing proton-exchange membranes (PEM) are described for NAD(P)H regeneration. In this setup, electrons obtained from water oxidation in the anode chamber are conducted to the cathode chamber through wires, where NAD(P)+ is reduced to NAD(P)H. The separation of chambers by PEM prevents oxygen produced in the anode from mixing with the cathode reactions. A reference electrode is placed in the working electrode chamber, which is part of a three-electrode system used in the process (Li Y et al., 2024; Bachar et al., 2025).

### 7.2 Cofactor economy at pilot and plant scale

Three credible NAD(P)H supply modes exist;

#### 7.2.1 Enzymatic recycling (GDH/FDH/PTDH)

GDH is widely utilized in early development due to its commercial availability, as shown by its frequent use in processes requiring cofactor recycling, with gluconic acid formation necessitating pH control and waste management. FDH is advantageous due to its minimal by-products, converting formate to CO_2_ which is easily removed and beneficial when minimizing mass intensity and by-product load is critical. The PSI-Fd-FNR system, while not a traditional enzymatic recycling method, enhances NADPH regeneration efficiency, as shown by its high turnover frequency. This offers an alternative approach for optimizing cofactor use in biocatalytic processes (France et al., 2023; Medipally et al., 2023).

#### 7.2.2 Electroenzymatic regeneration

Recent advances show high faradaic efficiency and strong 1,4-NAD(P)H selectivity using redox mediators/polymers, with continued improvements in current density and mediator recyclability. These systems decouple reducing power from sacrificial co-substrates and are well-suited for continuous operation in bioelectrochemical setups (Li Y et al., 2024; Jayakumar et al., 2025).

Hybrid electro-formate strategies that pair CO_2_-to-formate electrolyzers (approximately 90% FE at 150 mA cm^-2^ using Bi@C-600 electrodes) with [Cp*Rh(bpy)(H2O)]^2+^-catalyzed NADH regeneration offer a renewable-carbon route to electrons. This demonstrates high product titers and turnover numbers, with potential for CO_2_-neutral chemical processes when powered by renewable electricity (Wang C. et al., 2024).

#### 7.2.3 H_2_-driven biohydrogenation (cofactor-independent H_2_-driven biohydrogenation

Recently, it was reported that a [NiFe]-hydrogenase (Hyd-1) immobilised on carbon black to deliver electrons from 1 bar H_2_ directly to nitroarenes in aqueous buffer, achieved full conversion of 30 nitroarenes (isolated yields 78%–96%). The products included pharmaceuticals benzocaine, procainamide, and mesalazine, and 4-aminophenol, precursor to paracetamol. The catalyst is highly selective for nitro-group reduction over other unsaturated bonds, tolerant to a wide range of functional groups. This exhibits excellent stability in reactions lasting up to 72 h and full reusability over five cycles with a total turnover number over one million. Although not a nitroreductase process, the system completes the 6-electron reduction to amines under mild, precious-metal-free conditions and can be extended to aliphatic substrates using Hyd-2 (Sokolova et al., 2024).

The process-integration strategies described above have been successfully demonstrated on model nitroarenes in continuous packed-bed and electroenzymatic systems. Nevertheless, their application to nitro-sulfonamides remains to be explored. The proposed flowsheet presented here therefore serves as a plausible integration blueprint rather than a validated industrial process. Successful translation will require dedicated feasibility studies to confirm N-S bond integrity, hydroxylamine control, and overall performance under the specific physicochemical constraints of *p*-NBS.

**FIGURE 7:**
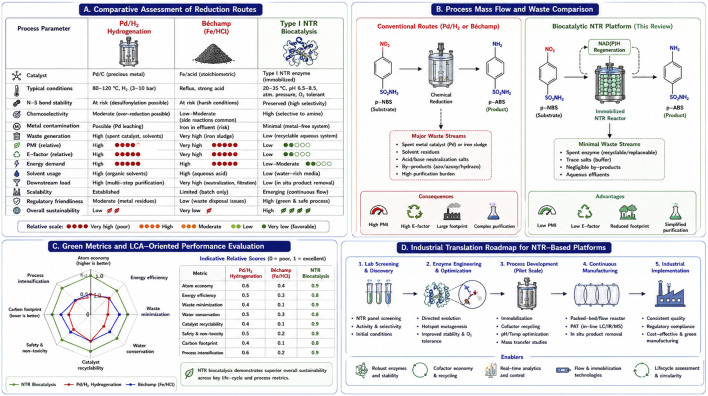
Conceptual sustainability and industrial translation framework for nitro-sulfonamide reduction routes.

Despite the strong conceptual basis for integrated Type I NTR-based nitro-sulfonamide biocatalysis, direct process-scale datasets for the *p*-NBS/*p*-ABS system remain limited. Important industrial parameters including substrate loading, long-term catalyst stability, volumetric productivity, isolated product yield, cofactor turnover efficiency, membrane extraction performance, process mass intensity (PMI), E-factor, energy demand, and comparative technoeconomic metrics versus conventional Pd/H_2_ hydrogenation have not yet been comprehensively reported for this specific substrate system. As a result, the proposed integrated reactor, cofactor-regeneration, analytical-monitoring, and sustainability frameworks presented in this review should presently be interpreted as forward-looking translational blueprints derived from established principles in nitroreductase catalysis, immobilized biocatalysis, continuous-flow processing, and green pharmaceutical manufacturing rather than as fully validated industrial process configurations. Figure 7 summarizes the comparative sustainability and industrial translation framework for conventional and biocatalytic nitro-sulfonamide reduction routes.

The conceptual process and sustainability frameworks highlight how future integration of selective Type I NTR catalysis with cofactor recycling, immobilized continuous-flow processing, and analytical process control could support development of scalable and environmentally sustainable nitro-sulfonamide manufacturing platforms following further substrate-specific validation.

### 7.3 O_2_ policy and mass transfer

NfsA, a Type I nitroreductase, performs obligatory two-electron reduction of nitroaromatic compounds (ArNO_2_) and quinones (Q), as evidenced by the absence of FMN semiquinone formation during 5-deazaflavin-sensitized photoreduction and a standard redox potential of FMN of −215 ± 5 mV at pH 7.0. This two-electron character, driven by hydride transfer, explains the lower reactivity of quinones compared to ArNO_2_ with equivalent single-electron reduction potentials (E_1_^7^). Computer modelling data reveal that H-bond formation with Arg15, Arg133, and Ser40 plays a major role in oxidant binding to reduced NfsA, while π–π interactions are less significant. Hydride-transfer distances are typically shorter for ArNO_2_ than for Q, and positively charged aliphatic substituents reduce reactivity. The reactivity of ArNO_2_ (except outliers nitracrine and SN-36506) increases with E_1_^7^ and is unaffected by lipophilicity or Van der Waals volume up to 386 Å^3^. Among quinones, 2-hydroxy-1,4-naphthoquinones are positive outliers with high reactivity and favorable activation entropy, whereas tetramethyl-1,4-benzoquinone is a negative outlier. All kinetic studies were conducted at pH 7.0 in 0.1 M phosphate buffer with 1.0 mM EDTA (Valiauga et al., 2024).

### 7.4 Process analytical technology (PAT) and specification setting

The product quality depends on consistent control of critical quality attributes (CQAs) during downstream purification steps. PAT must do more than track protein concentration. It should detect and quantify product-related variants (for example, size, charge, glycosylation) and confirm process endpoints across capture, wash, and elution phases. High-frequency, at-line/online LC is rapidly maturing and can be configured for milligram-to-kilogram monitoring. Real-time spectroscopic PAT (IR/UV) deconvolves component signals and flags excursions for automated feedback. Recent reports describe online LC architectures for real-time monitoring of critical quality attributes and robust PAT across downstream operations, and recent studies summarize the state-of-the-art PAT modalities applicable to biopharmaceutical purification (Graf et al., 2024; Sathiyapriyan et al., 2025).

### 7.5 Green metrics and LCA; measuring what matters

Early-stage decisions should be guided by E-factor and PMI alongside energy intensity but recent analysis of over 700 chemical processes demonstrate that mass- and energy-based metrics correlate only weakly with life cycle impacts. Therefore, LCA should be integrated with these process metrics to provide a more comprehensive environmental assessment (France et al., 2023; Lucas et al., 2024).

It has been reported that LCAs identify hidden hotspots (for example, solvent recovery, cold-chain, nitrogen use), ensuring that biocatalytic wins (lower metals, milder conditions) are not offset by upstream burdens (cofactor supply, buffer salts) (Chen et al., 2024).

Comparative LCAs on biocatalytic versus purely chemical routes for the complex cyclic dinucleotide 2′3′-cGAMP demonstrate that the enzymatic route significantly reduces toxicity-weighted impacts (human health: 5.9 × 10^−3^ DALY vs. 1.1 × 10^−1^ DALY, 19-fold lower) alongside all other assessed categories, including global warming potential (3,055.6 kg CO_2_ equiv. vs. 56454.0 kg CO_2_ equiv., 18-fold lower). This happens despite requiring more complex downstream processing due to aqueous dilution providing direct precedent for favouring biocatalytic steps in early-stage development of pharmaceutical CDNs (Becker et al., 2023).

### 7.6 Regulatory optics; elemental impurities and residuals

A strong sustainability argument for enzymatic routes is the elimination (or drastic reduction) of precious-metal residues, a major regulatory pain point governed by ICH Q3D. Current guidances (Q3D(R1)/Q3D(R2)) define route-specific PDEs for 24 elements. Notably, for iridium/rhodium/ruthenium/osmium there are insufficient data to set separate PDEs and palladium limits often apply by default, tightening process windows for many hydrogenations and mediator-assisted electroreduction schemes.

Enzyme routes do introduce proteinaceous impurities and potential DNA residuals when whole-cell catalysts are used but these are typically well managed by filtration and standard bioprocess polishing. They also lack the systemic toxicity profile of heavy metals, an advantage that is increasingly weighted in safety risk assessments.

### 7.7 Benchmarking against state-of-the-art chemocatalysis

Heterogeneous chemocatalysis continues to improve its sustainability credentials (for example, water as H source, non-precious catalysts, atomically precise clusters for product-selectivity steering), but sulfur-bearing substrates and sulfonamide motifs can still stress catalyst lifetimes and selectivity. The existence of robust, aqueous, low-pressure bio-routes provides a complementary pathway that avoids metal recovery and aligns with solvent minimization strategies (Ruan et al., 2024; Lai et al., 2025).

Strategically, organizations should develop a best available Pd/H_2_ fallback and an NTR-based primary route, letting green metrics, PAT-quantified impurity risk, and LCA decide at the end of development.

### 7.8 An integration blueprint for *p*-aminobenzenesulfonamide (*p*-ABS)

A credible flowsheet, consistent with current evidence, is as follows: (i) immobilize a Type I NTR (wild-type or engineered NfsB-lineage) on a hydrophilic, covalent carrier with pore sizes that minimize diffusional gradients; (ii) operate a temperature-controlled packed-bed (20 °C–30 °C) at pH 7.0–7.5, moderate ionic strength, and ≤10% benign cosolvent to manage *p*-nitrobenzenesulfonamide solubility; (iii) feed reduced nicotinamide generated either by an FDH loop (formate → CO_2_) or an electroenzymatic skid, selected after a techno-economic/PMI screen; (iv) implement online LC (plus IR) to track nitroso/hydroxylamine/azo signals and close the loop on residence time and cofactor feed; (v) polish by adsorption/filtration and crystallize *p*-ABS from water-rich media to reduce solvent intensity; (vi) run controlled experiments comparing PMI/E-factor and perform a gate-to-gate LCA to quantify benefits versus a Pd/H_2_ benchmark. Each element in this blueprint is supported by recent studies in immobilization, continuous NTR operation, cofactor regeneration, and analytics (Russo et al., 2025; Cosgrove et al., 2023; Li Y et al., 2024; Robescu and Bavaro, 2025; Graf et al., 2024). The scalability framework is presented in Table 3.

The operational windows, sustainability targets, and process configurations summarized in this table are hypothesis-driven translational benchmarks inferred from related nitroarene biocatalysis and pharmaceutical process-engineering literature. These values should not be interpreted as experimentally validated operating parameters for the *p*-nitrobenzenesulfonamide (*p*-NBS) to *p*-aminobenzenesulfonamide (*p*-ABS) system, for which direct pilot-scale datasets remain limited.

Importantly, the quantitative parameters summarized across Tables 1–3 in this study originate predominantly from related nitroarene and nitroheterocycle systems rather than dedicated *p*-NBS/*p*-ABS studies. Therefore, metrics such as substrate loading, enzyme productivity, cofactor turnover, isolated yield, PMI, E-factor, and long-term operational stability should presently be interpreted as indicative translational benchmarks rather than experimentally validated values for nitro-sulfonamide manufacture. Dedicated substrate-specific process studies remain necessary to establish industrial feasibility with high confidence.

## 8 Conclusion

Biocatalysis offers a credible, chemoselective route for reducing nitro-sulfonamides where N-S bond preservation is extremely important and where metal residues, hydrogen handling, and waste intensity limit traditional Pd/H_2_ and Béchamp options. The most practical starting point is a Type I NTR (NfsA/NfsB family), with engineered NfsB variants as immediate platforms when deactivated substrates stall. To improve selectivity, pair immobilised NTRs in packed-bed flow with tight electron supply control and on-line LC/IR PAT to manage hydroxylamine build-up and suppress azo/azoxy pathways. We propose to choose cofactor economy to fit constraints: GDH for simplicity (accept pH control), FDH for atom economy (formate → CO_2_), or electro-NAD(P)H when continuous operation and minimal waste dominate. Where appropriate, evaluation of cofactor-independent H_2_-to-substrate platforms or photo-assists as complementary options should be considered.

For *p*-ABS, a near-term flowsheet is viable: immobilised NTR at pH 7–7.5 and 20 °C–30 °C; ≤10% benign co-solvent for solubility; FDH or electro-regeneration for NAD(P)H; membrane extraction and crystallisation from water-rich media; and concurrent PMI/E-factor tracking with gate-to-gate LCA versus a Pd/H_2_ benchmark. Future experimental studies must establish substrate-specific quantitative benchmarks including conversion, isolated yield, operational stability, cofactor economy, PMI/E-factor, and technoeconomic feasibility for the *p*-NBS/*p*-ABS system.

Certain important gaps and opportunities are: (i) direct kinetics and scope on sulfonamide-linked nitroarenes; (ii) structure-guided engineering targeted at N-S integrity; (iii) scalable immobilisation chemistries for NTRs in saline/co-solvent systems; and (iv) prospective LCA case studies to lock in sustainability gains early. Addressing these challenges will convert today’s compelling lab-scale evidence into robust, plant-ready sustainable pathways to sulfanilamide cores.
