# Supplementary material for: Biocatalytic reduction of nitro-sulfonamides: enzyme engineering, process integration, and sustainable pathways to p-aminobenzenesulfonamide
Source: Front Bioeng Biotechnol. 2026 Jul 20;14:1867973. doi: 10.3389/fbioe.2026.1867973 (PMC13429851; doi:10.3389/fbioe.2026.1867973)
Supplement: Supplementary file 1 [file Table1.docx]

**Supplementary Table S1.**

Representative nitroreductases and Old Yellow Enzymes (OYEs) reported for nitro-group reduction and discussed in this review article.

| **Enzyme / variant** | **Source organism** | **Enzyme class** | **Manuscript reference** | **UniProt accession to write** |
| --- | --- | --- | --- | --- |
| NfsA | *Escherichia coli* K-12 | Type I nitroreductase | 37, 39 | P17117 |
| NfsB | *Escherichia coli* K-12 | Type I nitroreductase | 39, 40 | P38489 |
| NfsB F70A/F108Y | Engineered *E. coli* NfsB | Type I NTR mutant | 40 | P38489 |
| *Enterobacter cloacae* nitroreductase / EcNR | *Enterobacter cloacae* | Oxygen-insensitive NTR | 11, 44 | Q01234 |
| NR-04 | Johnson Matthey nitroreductase collection | Nitroreductase | 23 | Not reported |
| NR-14 | Johnson Matthey nitroreductase collection | Nitroreductase | 23 | Not reported |
| NR-17 | Johnson Matthey nitroreductase collection | Nitroreductase | 23 | Not reported |
| NR-24 | Johnson Matthey nitroreductase collection | Nitroreductase | 23 | Not reported |
| NR-55 | Johnson Matthey nitroreductase collection | Nitroreductase | 47 | Not reported |
| BaNTR1 | *Bacillus amyloliquefaciens* | Nitroreductase | 44 | Not reported |
| GkOYE | *Geobacillus kaustophilus* | Old Yellow Enzyme | 14 | Q5KXG9 |
| GkOYE.8 | Engineered GkOYE | OYE mutant | 14 | Q5KXG9 |
| GkOYE.11 | Engineered GkOYE | OYE mutant | 14 | Q5KXG9 |
| GkOYE.13 | Engineered GkOYE | OYE mutant | 14 | Q5KXG9 |
| Classical OYE | Yeast OYE | Old Yellow Enzyme | 43 | [Q02899](https://www.uniprot.org/uniprotkb/Q02899/entry) |

UniProt accession numbers are provided where the enzyme source and sequence could be unambiguously identified from the primary literature or associated database records. Engineered variants are assigned the UniProt accession number of the corresponding parent enzyme because mutant proteins do not receive separate UniProt entries. For several screening-derived nitroreductases (NR-04, NR-14, NR-17, NR-24, and NR-55) and BaNTR1, accession information was not disclosed in the original source publications.

**Supplementary Table S2**

Representative engineered nitroreductase and OYE variants reported to exhibit enhanced catalytic or operational properties**.**

| **Variant** | **Parent enzyme** | **Mutations** | **Engineering strategy** | **Reported enhancement** | **Property improved** | **Ref.** |
| --- | --- | --- | --- | --- | --- | --- |
| NfsB F70A/F108Y | *E. coli* NfsB | F70A+F108Y | Active-site remodeling | Enhanced activity toward CB1954, metronidazole and related nitroaromatics | Catalytic efficiency / substrate scope | 40 |
| GkOYE.8 | GkOYE | Q102A+Y169F+R215A+R308A | Active-site engineering | Improved stereoselectivity in nitroalkene reduction | Selectivity | 14 |
| GkOYE.11 | GkOYE | Q102A+Y169F+R215A+R308A+G62W+D247I+H167A | Iterative mutagenesis | Enhanced activity and stereocontrol | Activity/selectivity | 14 |
| GkOYE.13 | GkOYE | Q102A+R215A+R308A+Y169W+G62W | Active-site engineering | Improved catalytic performance | Activity/selectivity | 14 |
| Encapsulated NfsB | NfsB | tdNfsB = Two copies of wild-type *E. coli* NfsB linked by a flexible linker | Nanocompartment encapsulation | Improved operational stability | Stability | 41 |
| Metagenome-derived NTRs | Various NTRs | None (21 natural variants from metagenome; no engineered mutations) | Functional discovery | Expanded substrate diversity | Substrate scope | 60,61 |
| ML-prioritized NTR variants | Various NTRs | ML-prioritized Multiple mutations identified computationally; exact positions vary between studies and were not uniformly reported. | Machine-learning-guided engineering | Accelerated identification of beneficial mutations | Engineering efficiency | 63 |
